# Mediastinal fibrosis as a late and fatal complication of treated tuberculosis mimicking a neoplastic process in a 34-year-old man

**DOI:** 10.1016/j.radcr.2023.09.001

**Published:** 2023-09-23

**Authors:** Manar Ezzahi, Zaid Ennasery, Sara El Malih, Amal Akammar, Nizar El Bouardi, Meriem Haloua, Moulay Youssef Alaoui Lamrani, Meryem Boubbou, Mounia Serraj, Mustapha Maaroufi, Mohamed Smahi, Amara Bouchra, Badreeddine Alami

**Affiliations:** a Department of Radiology and Interventional Imaging, CHU Hassan II Fez, Sidi Mohammed Ben Abdellah University, Fez, Morocco; b Department of Pneumology - CHU Hassan II Fez, Sidi Mohammed Ben Abdellah University, Fez, Morocco; c Department of Thoracic Surgery - CHU Hassan II Fez, Sidi Mohammed Ben Abdellah University, Fez, Morocco

**Keywords:** Fibrosis, Mediastinal, Tuberculosis, CT, MRI

## Abstract

Fibrosing mediastinitis, also known as sclerosing mediastinitis, is an uncommon condition marked by abnormal proliferation of fibrous tissue in the mediastinum. This condition may accrue either as an idiopathic condition or as a complication of an underlying disease process. Its pathogenesis remains unknown. However, most reported cases are incriminating abnormal immune-mediated hypersensitivity responses to Histoplasma infection. Other rare causes include tuberculosis, blastomycosis, and an idiopathic form that may be associated with other miscellaneous conditions. CT and MR imaging play a vital role in the diagnosis and management of this disease. We present a rare case of fibrosing mediastinitis as a late complication of tuberculosis in a 34-year-old man with a prior history of mediastinal tuberculosis, mimicking initially a neoplastic mediastinal process. We will describe this clinical case in the light of the literature and point out the contribution of radiological imaging in the diagnosis of this rare pathology.

## Introduction

Two forms of mediastinitis have been described in the literature, namely granulomatous fibrosing mediastinitis (FM) or focal FM, in which the fibrous tissue reaction is limited to the mediastinal lymph nodes, and diffuse FM, in which there is a more extensive reaction in the mediastinal space [1].

FM, also called sclerosing mediastinitis, is a rare disease characterized by dense mediastinal infiltrates that destroy normal fat levels. It is best detected by advanced imaging techniques such as computed tomography (CT) or magnetic resonance imaging (MRI) [2]. The exact cause and pathogenesis of FM are still unknown. In most cases, FM is thought to be due to an exaggerated host immune response to a previous granulomatous mediastinal infection. Most cases are associated with infection with Histoplasma capsulatum, which is common in southeastern, mid-Atlantic, and central United States [3]. Other infectious agents such as tuberculosis (TB), aspergillosis, blastomycosis, and cryptococcosis can also cause this condition [4]. Several examples of idiopathic forms of FM that may result from an autoimmune process have also been described, such as Behcet's disease, retroperitoneal fibrosis, orbital pseudotumors, and Riedel's sclerosing thyroiditis [5,6]. Tuberculosis is considered the second leading cause of FM, especially in nonendemic areas of histoplasmosis [4]. Although FM is a benign condition, it is often progressive and causes compression and obstruction of mediastinal structures. These structures include the tracheobronchial tree, esophagus, superior vena cava (SVC), and vessels and have been associated with high morbidity and mortality [7]. When universally accepted diagnostic criteria are lacking, the clinical diagnosis of FM becomes challenging. Nevertheless, CT and MR imaging are crucial in both diagnosing and treating this disease [8]. These imaging techniques play a key role in helping healthcare professionals identify and manage cases of FM effectively. The optimal therapeutic approach to FM remains controversial. Anecdotal case reports have suggested that some patients with FM may benefit from anti-inflammatory, antifungal, or antifibrotic drugs. However, similar results of surgical and nonsurgical procedures to relieve mediastinal compression vary from highly successful procedures to therapeutic failures associated with high peri- and postoperative mortality [[9], [10], [11], [12]]. We present a rare case of FM as a late complication of tuberculosis in a 34-year-old man with a history of mediastinal tuberculosis, we will describe this clinical experience in the light of literature findings, and highlight the role of radiographic imaging in the diagnosis and management of this rare pathology.

## Case presentation

The patient, B.M., is a 34-year-old Caucasian man who was referred to the emergency department of the university hospital center Hassan II of Fez for exploration of an episode of low abundance hemoptysis, with chronic progressive dyspnea and cough.

The patient has a prior history of mediastinal tuberculosis (4 years ago), that has been confirmed by mediastinal biopsy (mediastinoscopy) and histological study which was compatible with lymph node tuberculosis. Then the patient was treated successfully.

He has no other pathological medical or surgical history.

The patient reports, about a month ago, the appearance of a dry cough and progressive dyspnea with a single episode of hemoptysis, with anorexia and weight loss (about 5 kg in the last month), no night sweats, fever, or other extra-respiratory signs were reported.

Clinical examination upon admission revealed a well-orientated patient with mild cervical swelling and no significant distress. His vital signs were normal except for a respiratory rate of 23 breaths/min and oxygen saturation of 92% on room air at rest. Cardiovascular and respiratory examination showed many cervicothoracic collateral venous circulation and jugular venous distension with decreased breath sounds at auscultation. The rest of the examination was perfectly normal.

The patient was then admitted to the Pneumology department where he underwent a complete biological check-up, the results were as follows:

The complete blood count cell showed a WCC of 11800×10^9/L [Reference range: 4.5-11.00×10^9/L], a hemoglobin level of 12 g/L [Reference range: 10-15.5 g/dL], and a platelet count of 355×10^9 /L [Reference range: 150-400×10^9 g/dL]. C-reactive protein (CRP) was at 163 mg/L [Reference range < 4].

**Fig. 1:**
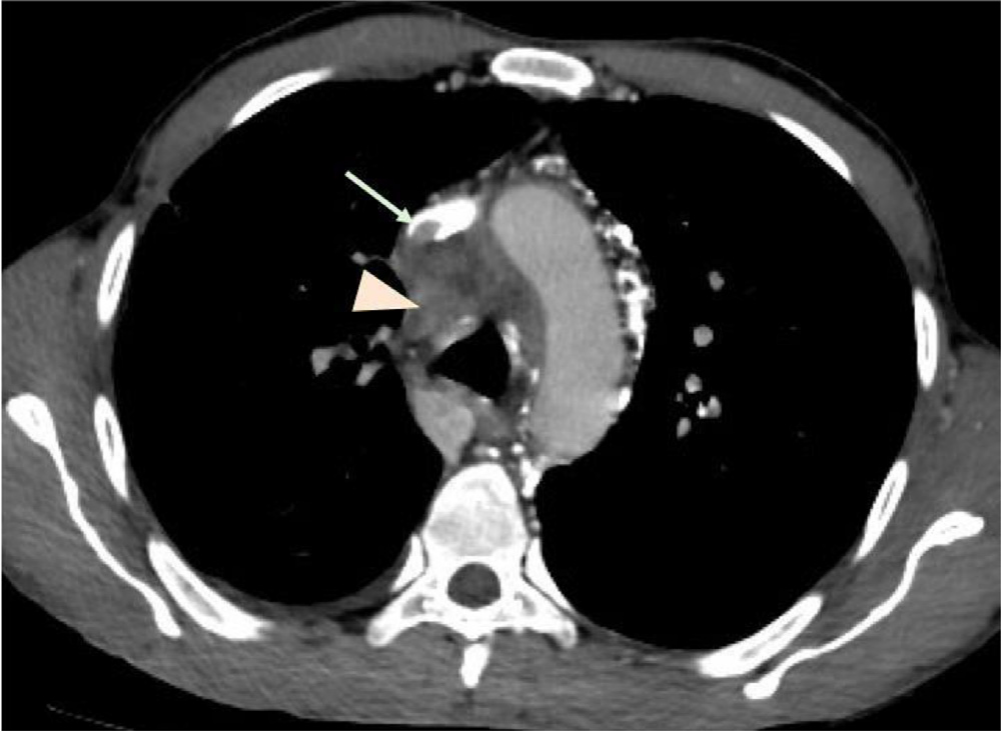
Axial contrast-enhanced CT image (soft-tissue window) showing infiltrative heterogeneous soft tissue (Arrowhead) encasing superior vena cava which is thrombosed (Arrow). We note also para-aortic collateral channels due to vena cava obliteration.

**Fig. 2:**
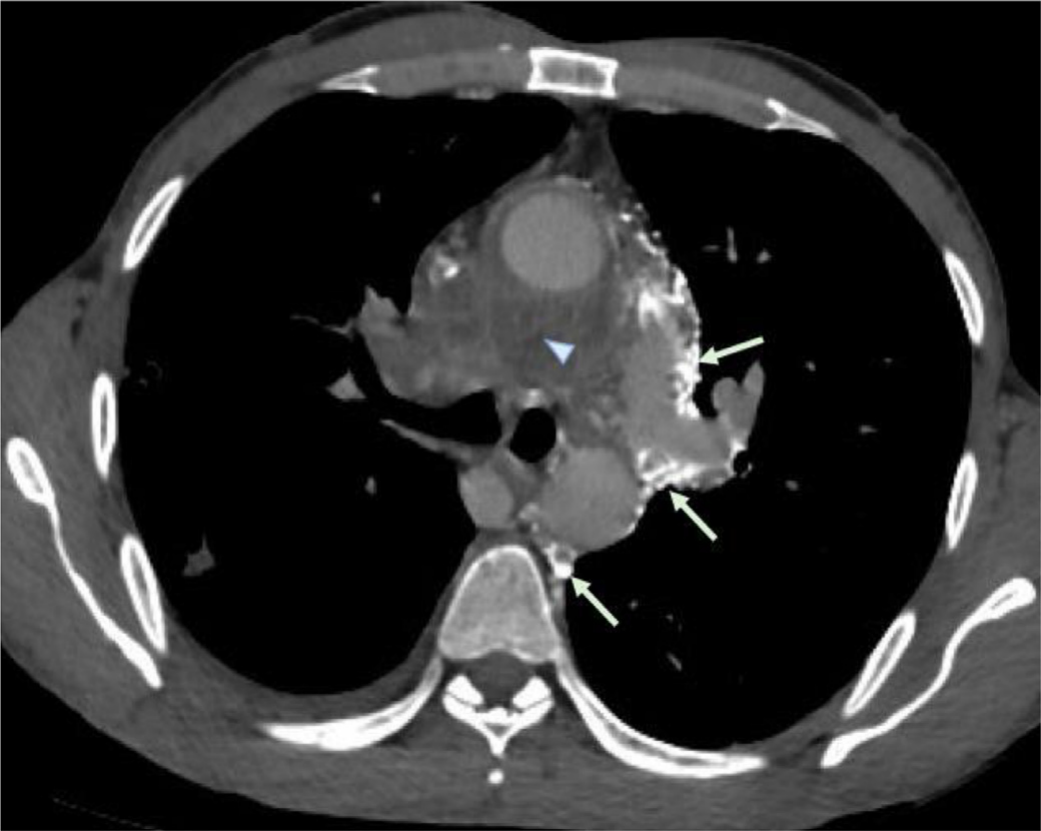
Axial contrast-enhanced CT image (soft-tissue window) showing the extension of the soft tissue into the aorto-pulmonary window, the pre- and sub-carinal spaces, and also in the right hilar region (arrowhead). We note also multiple collateral channels draining into the hemi-azygos vein which is early opacified (Arrows).

A thoracic enhanced CT scan was requested which showed, on the soft tissue window, a poorly limited mediastinal heterogeneous soft tissue, involving right paratracheal space, aorto-pulmonary window, pre and sub-carinal spaces and right hilar region (Figs. 1, 2, and 4).

**Fig. 3:**
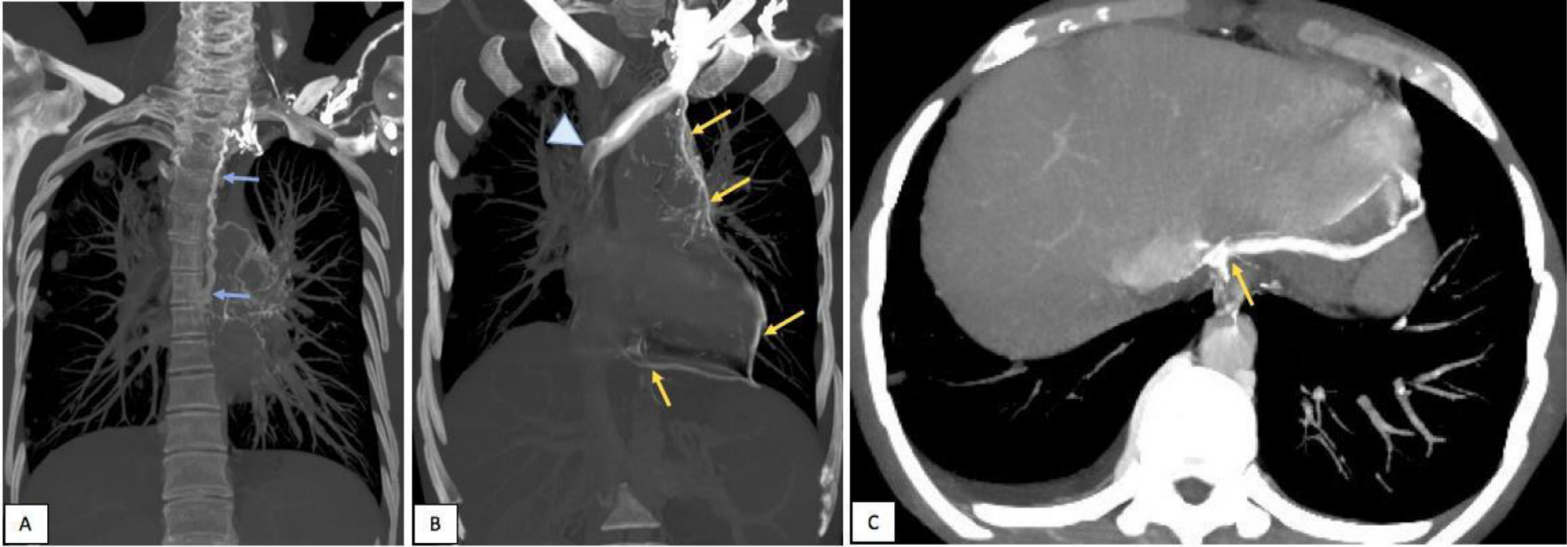
Coronal (A, B) and axial (C) contrast-enhanced CT image with MIP (maximal intensity projection) reconstructions (soft-tissue window); (A) collateral channels draining left brachio-cephalic vein into hemi- azygos which is enlarged (blue arrows). (B, C) collateral channels draining directly into the inferior vena cava (yellow arrows), with thrombosis and abrupt encasement of superior vena cava (Arrowhead).

**Fig. 4:**
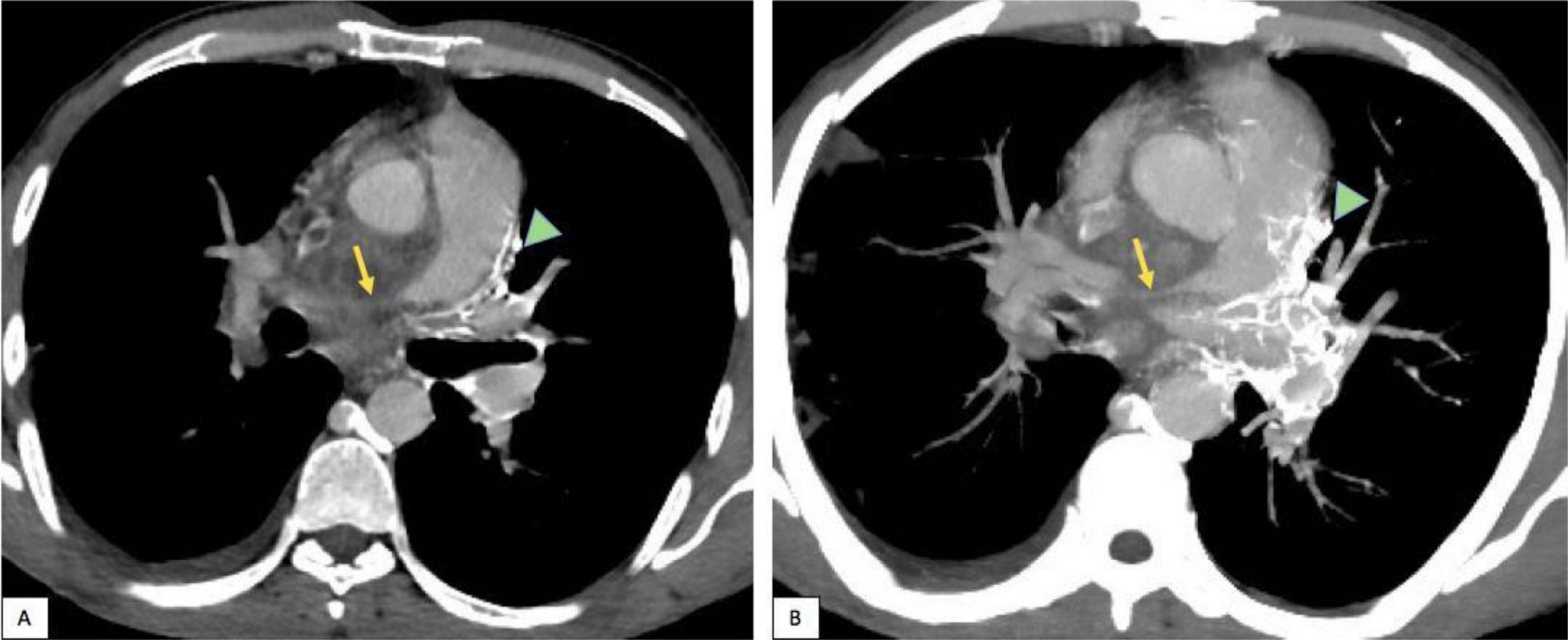
Axial contrast-enhanced CT images (soft-tissue window, before (A) then after MIP reconstruction (B), showing encasement and intense narrowing of the right pulmonary artery (arrows), with left mediastinal collateral channels (arrowhead). We note also a small thrombus in the lumen of the proximal right atrium (A).

This infiltrative soft tissue was encasing the SVC which was totally thrombosed (Figs. 1 and 3B) with a small extension of the thrombosis to the proximal right atrium and development of important collateral venous channels extending from the cervical region along the left side of the mediastinum and draining into the inferior vena cava (Fig. 1, Fig. 2, Fig. 3, Fig. 4).

This process was also encasing and narrowing the right pulmonary artery, and responsible for subtotal occlusion (Fig. 4).

**Fig. 5:**
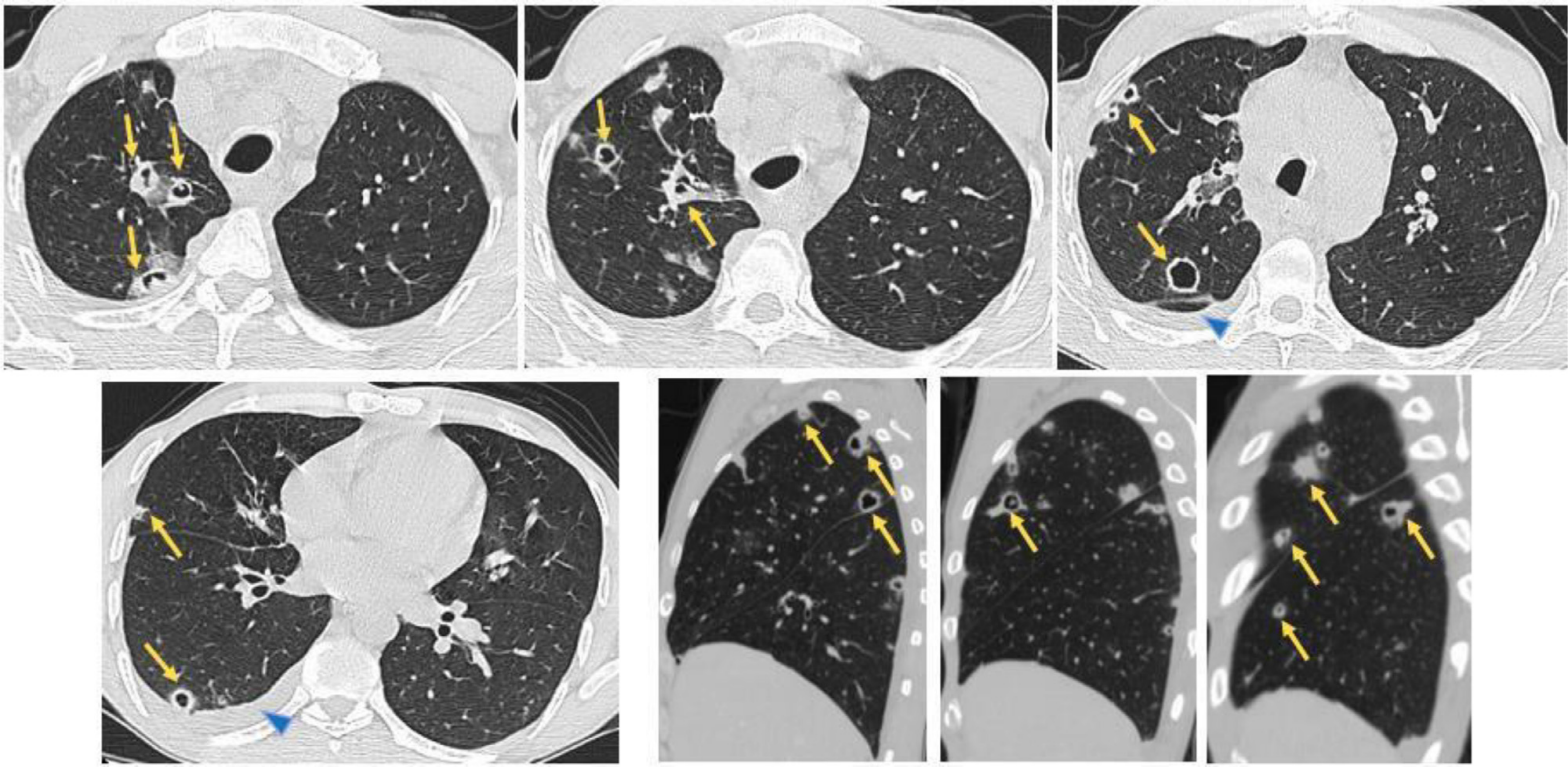
Axial contrast-enhanced CT images (lung window) show multiple right unilateral pulmonary nodules, of random distribution, of different shapes and sizes, excavated for the vast majority (Arrows), with a low abundance of right pleural effusion (Arrowhead).

On the lung window, we noticed the presence of right unilateral pulmonary nodules, of random distribution, of different shape and size, excavated for the vast majority, with a low abundance right pleural effusion (Fig. 5).

Thus we suggested first a neoplastic origin of the mediastinal mass and the right lung parenchymal lesions.

*Bronchoscopy* with broncho-alveolar lavage, brushings, and biopsies were performed. The bronchoscopy showed presence of a fistula at the anterior face of the trachea just above the carina, with thickened carina, without anomaly of the right and left bronchial trees.

The search for acid-alcohol resistant bacilli (BAAR) and GeneXpert test in the fibro-aspiration fluid were negative, and the histological study showed no malignancy.

Chest MRI was performed, some days later (1 month after the chest CT scan), for better characterization of the mediastinal mass.

**Fig. 6:**
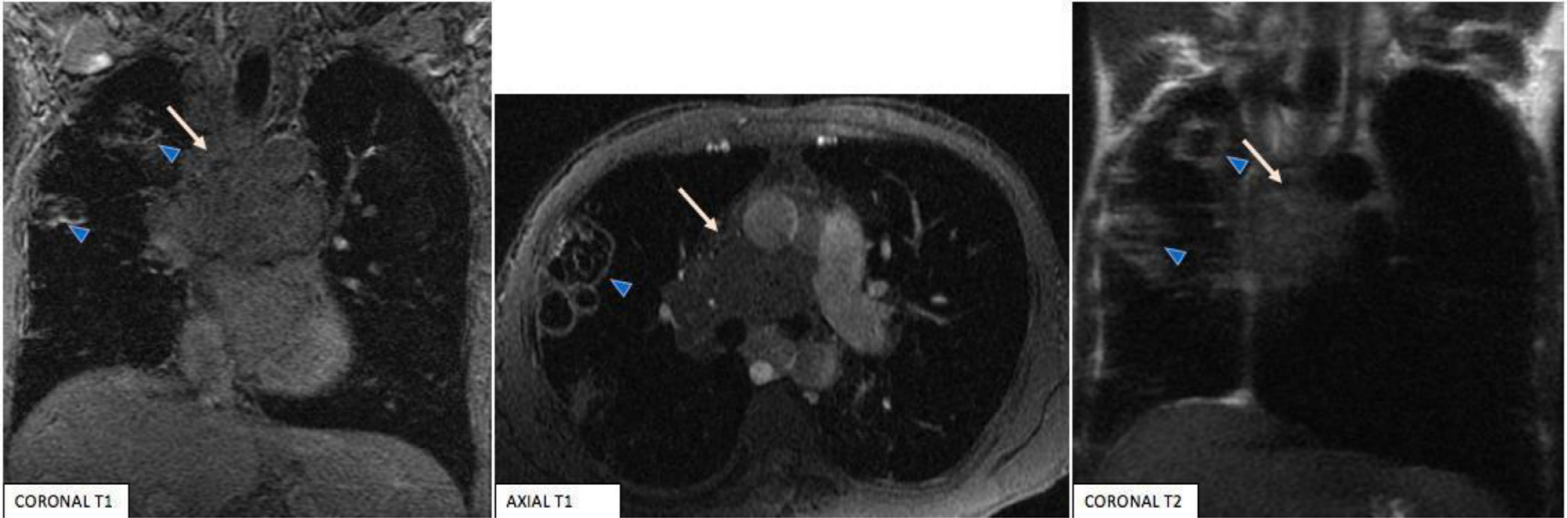
Chest MRI showing ill-defined infiltrative mediastinal fibrous soft tissue (Arrows), presenting low signal on T1 and T2-weighted images. We note also the pulmonary cavitary lesions (arrowheads).

The chest MRI showed an infiltrative mediastinal fibrous soft tissue, with ill-defined borders, which presented a low signal on T1 and T2-weighted images, no restricted diffusion, and poorly enhancement after contrast injection (Fig. 6). This process was encasing the pulmonary artery, the SVC, and narrowing the right main bronchus.

The MRI showed also an increase in size of the pulmonary parenchymal cavitary lesions (Fig. 6) described on the previous CT scan.

The patient's history of known mediastinal tuberculosis and the existence of old granulomatous disease on previous imaging (4 years ago), along with negative bronchoscopy findings, established *fibrosing mediastinitis* as the most probable cause of his mediastinal infiltrative soft tissue.

The evolution was marked some days later by the death of the patient due to respiratory distress.

## Discussion

FM is an uncommon medical condition characterized by an abnormal overgrowth of connective tissue and acellular collagen in the mediastinum. The exact cause and pathogenesis of FM are unknown, the most common hypothesis is that it is caused by a delayed hypersensitivity reaction to unknown fungi, mycobacteria, or other antigens. Histoplasmosis is considered the most common cause of fibrosing mediastinitis. However, most patients had pulmonary tuberculosis history prior to MF in regions where tuberculosis infection is common, notably in the Mediterranean region [4,13,14].

The prevalence of mediastinal fibrosis associated with tuberculosis is not known and only a few cases have been reported in the literature.

Most studies show a high incidence of granulomatous mediastinitis (localized form of FM) compared to the few cases of diffuse FM in which tuberculosis has been identified as the cause. The observed difference in TB incidence across the spectrum of granulomatous and fibrous disease may support the notion that the 2 conditions exist on a dynamic spectrum, with granulomatous mediastinitis occurring early in active infection when BAARs are more likely to be seen. Only a few cases of granulomatous mediastinitis lead to diffuse FM, where the fibrous tissue invades the rest of the mediastinum. Therefore, FM probably represents the later stages of an aberrant healing response after active infection. This theory is seen in many later case reports where fibrotic mediastinal tissue was culture negative for acid-fast bacillus (AFB) and TB, it is also seen in fibrosis associated with histoplasmosis where most cases are also culture-negative. However, it is important to note that due to the rarity of the disease, it is unclear in the literature whether these 2 forms of mediastinitis represent the ends of a spectrum or distinct clinical entities [15,16]. The early signs and symptoms of FM vary depending on the specific anatomical structures affected by the fibrotic reaction. The most commonly affected structure is the superior vena cava (SVC). Other areas include the esophagus, trachea, bronchi, pulmonary vein and artery, inferior vena cava, thoracic ducts, atrium, and recurrent laryngeal nerve. Although SVC obstruction is a common feature, the slowly progressive nature of this condition allows for the formation of collateral veins, and patients may often be initially asymptomatic. Progression of the disease can cause shortness of breath, swelling of the neck and face, and dilated chest veins. If bronchial structures are involved, progressive shortness of breath, coughing and bleeding may occur. Other complaints include dysphagia with esophageal invasion, hoarseness and recurrent laryngeal nerve entrapment, and nonspecific chest pain and malaise [1,4,15]. Several methods have been reported to obtain a diagnosis of FM. Clinical and laboratory findings are nonspecific, and transbronchial biopsies are often nondiagnostic. Although radiological methods suggest the diagnosis in most cases, thoracotomy and histopathological examination are still required for confirmation [17]. The sensitivity and specificity of plain X-ray in the diagnosis of fibrosing mediastinitis are low. Abnormal chest radiographs may reveal mediastinal or hilar masses that may contain calcifications, pulmonary infiltrates representing acute or chronic pneumonia due to bronchial obstruction, or pulmonary infarction due to vascular occlusion [13,18].

CT is currently the mainstay of imaging. CT findings in FM are well described and consist of a diffuse, infiltrative, or well-defined mediastinal mass, sometimes with compression of the tracheobronchial tree, large mediastinal vessels, or esophagus. In pulmonary venous obstruction, increased lung attenuation, interlobular septal thickening, and peri-bronchial obstruction can be observed. Calcification, which is patchy or dense, usually occurs diffusely throughout the mass [13,14,19]. MRI is comparable to CT in determining mediastinal involvement, but cannot consistently demonstrate calcification, which is a critical diagnostic finding in most cases of fibrosing mediastinitis. However, MRI has 2 distinct advantages over CT: it does not rely on intravenous contrast to demonstrate vascular progressive patency, and it can often reveal the fibrous nature of the mass. Although the signal intensity of mediastinal soft tissue masses varies in individual case reports describing the MR features of tuberculous fibrous mediastinitis, most authors describe hypointense areas on both T1 and T2-weighted images with late enhancement as characteristic of this disease [19,20,–21]. However, immature fibrosis, which contains little collagen and many vascular endothelial cells and fibroblasts, may have heterogeneous variable signal intensity on T2-weighted images [22,23].

Management of FM is mostly aimed at relieving symptoms, if any. There is currently no treatment that has been shown to prevent the effects of this granulomatous disease or alter its natural progression. Some studies show beneficial results with corticosteroids in the treatment of FM. Treatment of underlying infectious processes is still warranted for patient well-being and public health. Other treatment options may include percutaneous stenting of vascular structures such as the SVC to relieve obstruction or endoscopic stenting for airway obstruction and dysphagia. Surgery is of little benefit and generally results in poor outcomes due to calcifications, the extent of fibrosis, and extensive vascular collaterals. The natural development of FM includes the development of cardiopulmonary and airway damage due to recurrent infections, bronchial obstruction, or hemoptysis [3,11,13,24,25].

## Summary

Although uncommon, FM can be an unexpected sequel to successfully treated tuberculosis. It is a benign, slowly progressing inflammatory process leading to sclerosis around any mediastinal structure. The severity and range of symptoms experienced by a patient are directly proportional to the degree of fibrous tissue infiltration in the mediastinum. FM is a diagnosis of exclusion and is best diagnosed by advanced imaging modalities. It is most often a late sequel to granulomatous inflammation, but active processes such as infection or malignancy should be excluded initially. The management is mainly aimed at relieving symptoms, if present, especially since no medical treatment or surgery has proven to be beneficial.

## Ethics approval and consent to participate

Not applicable.

## Availability of data and materials

The data sets are generated on the data system of the CHU Hassan II of Fes, including the biological data and the interventional report.

## Funding

No source of funding was received.

## Patient consent

Written informed consent was obtained from the patient for publication of this case report and any accompanying images. A copy of the written consent is available for review by the Editor-in-Chief of this journal.
